# State-level income inequality and mortality among infants born in the United States 2007–2010: A Cohort Study

**DOI:** 10.1186/s12889-019-7651-y

**Published:** 2019-10-22

**Authors:** Roman Pabayo, Daniel M. Cook, Guy Harling, Anastasia Gunawan, Natalie A. Rosenquist, Peter Muennig

**Affiliations:** 1 University of Alberta, School of Public Health, 3-300 Edmonton Clinic Health Academy, 11405-87 Ave, Edmonton, Alberta T6G 1C9 Canada; 2 University of Nevada, Reno, School of Community Health Sciences, 1664 N Virginia St., Reno, 89557 USA; 3 Institute for Global Health, University College London, London, WC1E 6BT UK; 4 Mailman School of Public Health, Columbia University, 722 West 168th St. 4th Floor, New York, NY 10032 USA

**Keywords:** Social determinants of health, Social epidemiology, Income inequality, Infant mortality

## Abstract

**Background:**

United States state-level income inequality is positively associated with infant mortality in ecological studies. We exploit spatiotemporal variations in a large dataset containing individual-level data to conduct a cohort study and to investigate whether current income inequality and increases in income inequality are associated with infant and neonatal mortality risk over the period of the 2007–2010 Great Recession in the United States.

**Methods:**

We used data on 16,145,716 infants and their mothers from the 2007–2010 United States Statistics Linked Infant Birth and Death Records. Multilevel logistic regression was used to determine whether 1) US state-level income inequality, as measured by Z-transformed Gini coefficients in the year of birth and 2) change in Gini coefficient between 1990 and year of birth (2007–2010), predicted infant or neonatal mortality. Our analyses adjusted for both individual and state-level covariates.

**Results:**

From 2007 to 2010 there were 98,002 infant deaths: an infant mortality rate of 6.07 infant deaths per 1000 live births. When controlling for state and individual level characteristics, there was no significant relationship between Gini Z-score and infant mortality risk. However, the observed increase in the Gini Z-score was associated with a small but significant increase likelihood of infant mortality (AOR = 1.03 to 1.06 from 2007 to 2010). Similar findings were observed when the neonatal mortality was the outcome (AOR = 1.05 to 1.13 from 2007 to 2010).

**Conclusions:**

Infants born in states with greater changes in income inequality between 1990 and 2007 to 2010 experienced a greater likelihood of infant and neonatal mortality.

## Background

The infant mortality rate (IMR), defined as the number of infant deaths before the age of 1 year per 1000 live births, is one of the most important and powerful measures of a nation’s life expectancy [1]. Infant mortality is associated with the economic and social conditions that shape the health of mothers and newborns. These economic and social conditions include individual characteristics, such as the home environment and lifestyles. They also include state-level characteristics, such as the quality and availability of medical care within the local health system [2]. In 2011, around 24,000 infants died in the United States (US), resulting in an IMR of 6.1 [3].

This rate is higher than the Organization for Economic Co-Operation and Development (OECD) average of four deaths per 1000 births [2]. In comparison to other nations of similar (or even lower) per capita income, the US IMR is notably higher. For example, in 2016, the IMR in Japan was 2.3, and most western European nations had IMRs well under 4. The US IMR is more comparable to (or even higher than) rates in eastern Europe (e.g., Slovak Republic, Latvia, Russia) but has a lower rate than in upper-middle-income countries such as Chile, Turkey or Mexico [4].

In the US, there are differences in IMR’s by sociodemographic characteristics such as mother’s age, race/ethnicity, and socioeconomic status. For example, the 2010 IMR among non-Hispanic black, Hispanics, and American Indian and Alaskan Native was 11.48, 5.25, and 8.28, respectively, which were higher than for non-Hispanic whites (IMR = 5.18) [3]. Also, the IMRs among immigrants are lower than that among US-born mothers and infants [3].

Income inequality might also be a proxy for other US state-level characteristics that are determinants of infant mortality. US states that have unequal distributions in income may be less likely to invest in quality education and health care [5]. State governments that distribute incomes more equitably do so by investing in social goods, which can have a beneficial impact on maternal and infant health [5]. Therefore, earlier research on the United States has focused on state-level income inequality and a variety of health outcomes (including infant mortality) [6–8]. However, due to the lack of available individual-level data, this work tended not to examine the relationship between state-level income inequality and individual-level risk.

Socioeconomic characteristics and social conditions have proven to be significant risk factors for IMR. For example, poverty can potentially lead to conditions that are detrimental to maternal and infant health [6]. This is defined as the *absolute income* hypothesis, which specifies that an individual’s health depends on their own level of income [9]. Low income, or low socioeconomic status, has proven to be a significant risk factor for more immediate causes of infant mortality such as serious birth defects, preterm birth before 37-weeks’ gestation, Sudden Infant Death Syndrome (SIDS), maternal complications of pregnancy and injury [10]. Thus, poverty (a measure of absolute income) is a fairly well established risk factor for infant mortality.

The *relative income* hypothesis states that health depends not solely on one’s own income, but also on the incomes of others in society [9]. Since the late 1980’s and early 1990’s, there has been growing income inequality within OECD countries [11]. The relative distribution of incomes in society, and increasing gap between rich and poor, has been hypothesized to influence an individual’s risk for health and well-being, independent of an individual’s own income level, in part by creating social stress [12]. Pickett & Wilkinson [12] contend that when the gap between the incomes of the wealthy and poor widens, it might heighten feelings of insecurity and shame among members of society who are left behind. This is known as the so-called “psychosocial theory” of income inequality and health. Also, researchers have argued that unequal societies are damaging to the health of everybody in that society, including the wealthy, because income inequality erodes social cohesion. A loss in social cohesion is hypothesized to lead to social exclusion, social isolation, the loss of public goods, such as education, health care, and public infrastructure [13]. These factors can have an influence on the infant mortality risk (via psychological stress and material hardship) as well as neonatal (infants aged under 28 days) mortality risk (via reduced access to health care). Multilevel analyses have demonstrated that there is an excess risk of morbidity and mortality associated with living in a society with high levels of income inequality, even after adjustment for the confounding effects of individual income [7]. Nonetheless, other epidemiologists are not convinced that income inequality is a major, and generalizable determinant for adverse health outcomes [14]. They argue that within the US, it is education and state-level policies toward the poor, that are correlated with income inequality, that explains the relationship between income inequality and adverse health outcomes [14].

Among these adverse health outcomes, researchers observed a relationship between income inequality and IMR ([8, 15–21]. For example, in the US, states with high-income inequality had higher IMR [15, 16]. Income inequality is also significantly associated with other indicators of infant health [22]. State income inequality was positively correlated with preterm birth rate, and low and very low birthweight [16].

A few studies have explored the relationship between contextual income inequality and infant health outcomes on the individual level, i.e. using disaggregated outcome data. Earlier work showed that, in Japan, mothers who lived in prefectures with middle and high Gini coefficients were more likely to deliver a small-for gestational age infant in comparison to mothers living in prefectures with lower Gini coefficients [23]. In the US, the relationship between state-level economic inequality and an infant’s probability of death was examined using data from the 1985, 1987, and 1991 US Vital Statistics Linked Birth and Death Records. Those researchers found that state-level economic inequality is associated with higher odds for neonatal mortality, but not post-neonatal mortality, after adjusting for mother’s age, race, and state characteristics [22]. The same study suggested that economic segregation and state health care spending acted as mechanisms through which income inequality is associated with odds for neonatal mortality [22].

However, processes such as gentrification unfold over time. To date, associational studies have failed to account for changes over time, increasing the likelihood that the findings are spurious [24]. For one, wealthy people demand (and pay for) higher quality public goods, like hospitals, schools, or grocery stores. However, these institutions take time to infiltrate and mature in areas that are becoming wealthier and also tend to disappear slowly in areas that are becoming poorer. Because cross-sectional studies do not account for changes in inequality over time, they do not capture changes in health over time. An area that is gentrifying can appear to have no change in health impacts for the average person if health is increasing among the wealthy but declining among the poor.

We explore race as an effect modifier because contextual factors might have a differential association with health outcomes across socio-demographic groups, such as race [25, 26], and SES [27]. This differential impact across socio-demographic groups might be observed with infant and maternal health outcomes, and might explain why infant mortality rates are higher among non-Hispanic blacks in comparison to non-Hispanic whites. Recent evidence indicates that income inequality is related to an increased risk for death among non-Hispanic black adults, in comparison to non-Hispanic whites [28]. For example, using census data, researchers observed that for each unit increase in income inequality, there were an additional 27 to 37 additional deaths per year among non-Hispanic black adults. Among non-Hispanic white Americans, each unit increase in income inequality resulted in 417 to 480 fewer deaths per year [28]. Therefore, future analyses should test individual socio-demographic characteristics and contextual cross-level interactions. Finally, a critical but often overlooked piece of any temporal analysis that includes both SES and health is nativity. In the US, the foreign-born are in much better heath than the native-born, but tend to have lower incomes [29].

The objectives of this study are not only to identify the relationship between income inequality and odds for infant and neonatal mortality but to study the effects of change in income inequality, while adjusting for potential confounders at both the individual and state level. This work aims to extend the existing US ecological literature on state-level income inequality and infant mortality to look at individual-level outcomes through a multilevel lens. To fully capture temporal effects, we focus on the time span over the Great Recession of the early 1990s, a time when income inequality increased dramatically.

## Methods

### Data source

Data were obtained from the U.S. Cohort Linked Birth/Infant Death Data Files 2007–2010, which is provided by the National Center for Health Statistics (NCHS). In the United States, state laws require birth certificates to be completed for all births, and federal law mandates national collection and publication of births and other vital statistics. The National Vital Statistics System compiles these data and is the result of the cooperation between NCHS and the states. LBID contains information on maternal and infant socio-demographic characteristics, place of birth, and risk factors for infant health. In the 2007–2010 data, over 98% of death records were linked to the corresponding birth certificate. We excluded those missing demographic information, and foreign residents or those with records that indicated mismatch between state of birth and mother’s state residence. Infants were followed until their first birthday. Ethical approval was obtained from the University of Nevada, Reno Institutional Review Board.

### Measures

Our two outcomes were infant mortality, death within 365 days of birth, and neonatal mortality, death within the first 28 days of life. Our primary exposure was state income inequality, measured by the Gini coefficient of 2007 to 2010. For this investigation, the pre-tax income was used. The Gini coefficient ranges from 0 (perfect equality, where every household earns exactly the same income) to 1.0 (perfect inequality, where one household has all the income in the state). The calculation of the Gini coefficient has been described elsewhere [15]. For this investigation, the Gini coefficient in each of the 50 states and the District of Columbia was obtained from 2007 to 2010 from the US Census. We additionally calculated the change in Gini coefficient from 1990 to the year of birth. Both the Gini coefficients and change in Gini coefficients were z-transformed or standardized for analysis.

The state-level factors we controlled for include: 1) median income. Because wealthier states (e.g. California, New York) tend also to have more generous, rational, and high quality welfare policies, there is a good deal of collinearity between median income and quality welfare provision; 2) proportion of the population that is non-Hispanic black. States that have high proportions of non-Hispanic black (e.g., Alabama, Georgia) also tend to have weaker welfare provision. Part of the reason for this may be racial discrimination, which can also contribute to program quality; 3) population size. States with larger populations (e.g., California and New York) tend to have more generous and high quality welfare provision; and 4) which of the nine geographic US census divisions the state is in. This variable is intended to control for the remaining variation in welfare provision and equality by ensuring that large states with a somewhat higher median income but lower welfare provision (e.g., Texas) are accounted for. Additionally, in all analyses we included individual-level maternal covariates at infant birth: mother’s age, race/ethnicity, education, marital status, and nativity (US vs. foreign born).

### Statistical analysis

Infants with missing information were excluded from the analyses. We used multilevel logistic modeling (mothers and their infants nested within states) to determine the association between state-level income inequality and infant and neonatal mortality.

To investigate the potential effect of income inequality and change in income inequality and likelihood of infant mortality, we adopted a step-up approach and conducted three sets of analyses [30]. We first estimated the null model to compute the 95% plausible value range, which is an indication of the degree of variability of the likelihood of infant mortality and neonatal mortality. For example, the plausible value range allows us to compute a range of plausible proportion experiencing infant and neonatal mortality across the US states. Second, we conducted analyses including state-level and individual-level socio-economic characteristics, including income inequality. Finally, to determine whether an association between Gini coefficient or change in Gini coefficient and infant mortality differed across racial groups and mother’s nativity status, we repeated our analyses separately among non-Hispanic white and non-Hispanic black mothers and US-born and foreign born mothers and their infants.

## Results

Between 2007 and 2010, there were 16,145,716 births. Data for 16,346,267 / 16,724,137 infants = 97.7% born in 2007 to 2010 was included in our analysis. Those who had missing data were more likely to have mothers who were: Asian and Hispanic than non-Hispanic white; foreign-born; single; older; and to be from the Pacific and Mountain census divisions. In comparison to infants who were first born, those excluded were also less likely to be second or third born, but more likely to be fourth born or more.

Characteristics of the infants and their mothers can be found in Table 1. Just over half of the mothers were non-Hispanic white (53.5%), a quarter was Hispanic (24.5%), and 15 % were non-Hispanic black. Over one half of the mothers (52.0%) had a post-secondary education, and a majority were married (59.3%) and were US born (76.1%). State-level characteristics are also found in Table 1. In 2010, the average Gini coefficient was 0.45 (SD = 0.20). The average change in Gini coefficient from 1990 to 2010 was 0.06 (SD = 0.01). The median state-level income was $49,973.65 (SD = 8130.60) and the average proportion of non-Hispanic blacks was 12% (SD = 11.8).

**Table 1:** Characteristics of mothers and US infants born 2007–2010

Individual Level Characteristics	n	Percentage
Birth year		
2007	4,173,087	25.9
2008	4,109,463	25.5
2009	3,993,282	24.7
2010	3,869,884	24.0
Mother’s Race		
non-Hispanic white	8,640,230	53.5
non-Hispanic black	2,383,748	14.8
American Indian	159,375	1.0
Asian	927,957	2.7
Hispanic	3,960,440	24.5
Other	73,965	0.5
Education		
Less than high school	3,288,457	20.4
High School	4,468,279	27.7
Post-secondary	8,388,980	52.0
Marital Status		
Single	6,579,444	40.8
Married	9,566,272	59.3
Birth order		
First	5,378,396	33.3
Second	4,539,641	28.1
Third	2,918,817	18.1
Fourth or more	3,308,862	20.5
Nativity		
Foreign-born	3,855,491	23.9
US Born	12,290,225	76.1
US State-Level Characteristics 2010	Mean (SD)	Min, Max
Gini	0.45 (0.20)	(0.42, 0.53)
Change in Gini 1990–2010	0.06 (0.01)	(0.04, 0.10)
Median Income, USD	49,973.65 (8130.60)	(36,851, 68,854)
Population, 2010	6,054,080 (6824211)	(563,767, 37,300,000)
non-Hispanic black (%)	12 (11.8)	(0.40, 0.55)
Proportion in poverty (%)	14.8 (3.1)	(8.3, 22.4)

During 2007 to 2010, there were 98,002 infant deaths, leading to an infant mortality rate of 6.07 infant deaths per 1000 births. Of these deaths, 63,086 were neonatal deaths, or a neonatal mortality rate of 3.91 neonatal deaths per 1000 births. For every year 2007–2010, the overall predicted probability of observing infant deaths and neonatal deaths across US states and the plausible value range were calculated from the intercept model are found in Table 2. In 2007, the overall predicted probability was 0.64 and 0.41% for infant and neonatal mortality, respectively. The overall predicted probability slightly decreased every year from 2007 to 2010. The plausible value range indicates that there is considerable variability in the cumulative incidence of infant and neonatal deaths across the US states (Table 2). The plausible value range for neonatal mortality and infant mortality in 2007 was respectively 0.29–0.58% and 0.46–0.90%; by 2010 these ranges were 0.26–0.52% and 0.40–0.80%.

**Table 2:** Neonatal Mortality and Infant Mortality Overall Predicted Probability of death and Plausible Value Range Across US states 2007–2010

	Overall Predicted Probability of death (Plausible Value Range Across US states) 2007-2010			
Outcome	2007	2008	2009	2010
Neonatal mortality	0.41 (0.29–0.58)	0.39 (0.28–0.55)	0.38 (0.27–0.54)	0.37 (0.26–0.52)
Infant mortality	0.64 (0.46–0.90)	0.61 (0.44–0.86)	0.60 (0.43–0.84)	0.57 (0.40–0.80)

When we tested the crude relationship between state-level income inequality and change in income inequality and the outcomes infant mortality and neonatal mortality, from 2007 to 2010, state-level income inequality, but not change in income inequality was associated with both infant mortality and neonatal mortality. For every increase in standard deviation of Gini coefficient, there was a significant increase in odds both infant mortality (Table 3) and neonatal mortality (Table 4).

**Table 3:** The relationship between income inequality and infant mortality controlling for individual and state-level characteristics

	2007				2008				2009				2010			
	Odds for Infant Mortality				Odds for Infant Mortality				Odds for Infant Mortality				Odds for Infant Mortality			
	Crude		Adjusted		Crude		Adjusted		Crude		Adjusted		Crude		Adjusted	
	OR	95%CI	OR	95%CI	OR	95%CI	OR	95%CI	OR	95% CI	OR	95%CI	OR	95%CI	OR	95%CI
State characteristics																
Gini Z-score	1.11	(1.05,1.18)	1.01	(1.00,1.07)	1.06	(1.00,1.13)	0.98	(0.94,1.01)	1.10	(1.04,1.17)	1.01	(0.95,1.08)	1.09	(1.10,1.16)	1.04	(0.97,1.11)
Change in Gini Z-score	0.94	(0.93,0.96)	1.03	(1.00,1.07)	0.96	(0.91,1.02)	1.04	(1.01,1.08)	0.94	(0.89,0.99)	1.03	(0.98,1.08)	0.94	(0.89,0.99)	1.06	(1.00,1.12)
State Median Income Z-score			0.91	(0.87,0.95)			0.92	(0.87,0.96)			0.96	(0.88,1.04)			0.82	(0.78,0.87)
Proportion non-Hispanic black Z-score			1.01	(0.97,1.05)			0.99	(0.95,1.03)			0.98	(0.93,1.03)			0.96	(0.90,1.03)
Proportion Poor Z-score			0.91	(0.86,0.97)			0.92	(0.87,0.98)			0.99	(0.89,1.09)			0.94	(0.83,1.06)
State Population Z-score			0.99	(0.97,1.01)			1.00	(0.98,1.02)			0.98	(0.95,1.01)			0.99	(0.96,1.03)
Census Division (ref: New Engl)																
Middle Atlantic			0.97	(0.89,1.06)			1.03	(0.94,1.14)			1.00	(0.87,1.15)			1.13	(0.97,1.32)
East North Central			1.16	(1.05,1.28)			1.19	(1.06,1.33)			1.21	(1.04,1.40)			1.34	(1.13,1.59)
West North Central			1.11	(1.00,1.23)			1.14	(1.01,1.28)			1.03	(0.89,1.19)			1.19	(1.00,1.42)
South Atlantic			1.17	(1.05,1.31)			1.22	(1.08,1.38)			1.18	(1.00,1.38)			1.36	(1.11,1.65)
East South Central			1.29	(1.15,1.45)			1.38	(1.22,1.56)			1.23	(1.02,1.48)			1.52	(1.22,1.89)
West South Central			1.19	(1.07,1.33)			1.23	(1.09,1.39)			1.18	(0.99,1.40)			1.43	(1.15,1.78)
Mountain			1.20	(1.07,1.35)			1.13	(0.99,1.28)			1.14	(0.96,1.36)			1.25	(1.04,1.52)
Pacific			1.07	(0.96,1.19)			1.07	(0.95,1.21)			1.02	(0.86,1.21)			1.12	(0.93,1.35)
Individual Characteristics																
Mother’s Age (years)			1.00	(0.99,1.00)			0.99	(0.99,1.00)			0.99	(0.99,1.00)			1.00	(0.99,1.01)
Mother’s Race (ref: non-Hispanic white)																
non-Hispanic black			1.91	(1.85,1.98)			1.88	(1.82,1.95)			1.90	(1.84,1.97)			1.83	(1,77,1.90)
American Indian			1.35	(1.21,1.50)			1.23	(1.10,1.38)			1.38	(1.24,1.55)			1.30	(1.16,1.47)
Asian or Pacific Islander			1.16	(1.08,1.25)			1.13	(1.05,1.22)			1.16	(1.08,1.26)			1.14	(1.06,1.23)
Hispanic			0.97	(0.93,1.01)			0.98	(0.94,1.03)			0.96	(0.92,1.01)			0.98	(0.93,1.02)
Education (ref: less than HS)																
High School			0.90	(0.87,0.93)			0.88	(0.85,0.91)			0.88	(0.85,0.91)			0.91	(0.87,0.94)
Post-Secondary			0.68	(0.65,0.70)			0.67	(0.65,0.70)			0.67	(0.64,0.69)			0.68	(0.66,0.71)
Marital Status (ref: coupled)																
Single			1.35	(1.31,1.39)			1.31	(1.27,1.35)			1.32	(1.28,1.36)			1.34	(1.30,1.38)
Nativity (ref: born outside USA)																
US born			1.26	(1.21,1.31)			1.29	(1.24,1.35)			1.32	(1.27,1.38)			1.29	(1.24,1.35)
Birth Order (ref: first)																
Second			1.01	(0.98,1.04)			1.03	(1.00,1.08)			1.04	(1.00,1.07)			1.03	(0.99,1.07)
Third			1.06	(1.02,1.11)			1.12	(1.08,1.16)			1.10	(1.06,1.15)			1.10	(1.33,1.44)
Fourth or more			1.33	(1.28,1.38)			1.41	(1.35,1.46)			1.36	(1.31,1.42)			1.38	(1.33,1.44)

**Table 4:** The relationship between income inequality and neonatal mortality controlling for individual and state-level characteristics

	2007				2008				2009				2010			
	Odds for Neonatal Mortality				Odds for Neonatal Mortality				Odds for Neonatal Mortality				Odds for Neonatal Mortality			
	Crude		Adjusted		Crude		Adjusted		Crude		Adjusted		Crude		Adjusted	
	OR	95% CI	OR	95%CI	OR	95% CI	OR	95%CI	OR	95%CI	OR	95%CI	OR	95%CI	OR	95%CI
State Characteristics																
Gini Z-Score	1.10	(1.03,1.17)	0.99	(0.94,1.04)	1.05	(0.99,1.12)	0.95	(0.91,1.00)	1.10	(1.04,1.17)	0.95	(0.91,1.00)	1.11	(1.04,1.18)	0.96	(0.92,1.01)
Change in Gini Z-score	0.97	(0.92,1.03)	1.05	(1.01,1.09)	1.01	(0.95,1.07)	1.07	(1.03,1.11)	0.99	(0.93,1.04)	1.07	(1.03,1.12)	1.00	(0.94,1.06)	1.13	(1.07,1.19)
State Median Income Z-score			0.93	(0.87,0.98)			0.94	(0.88,1.00)			0.88	(0.82,0.94)			0.80	(0.74,0.87)
Proportion non-Hispanic black Z-score			1.02	(0.97,1.07)			0.99	(0.94,1.04)			1.04	(0.98,1.09)			1.04	(0.98,1.10)
Proportion Poor Z-score			0.94	(0.87,1.02)			0.95	(0.88,1.04)			0.92	(0.85,1.00)			0.82	(0.75,0.90)
State Population Z-score			0.99	(0.97,1.02)			1.01	(0.98,1.04)			1.02	(0.99,1.05)			1.06	(1.03,1.10)
Census Division (ref: New Engl)																
Middle Atlantic			0.89	(0.79,0.99)			0.93	(0.83,1.05)			0.84	(0.74,0.95)			1.01	(0.88,1.15)
East North Central			1.12	(0.99,1.28)			1.08	(0.94,1.24)			1.00	(0.88,1.15)			1.19	(1.01,1.40)
West North Central			1.12	(0.98,1.28)			1.01	(0.87,1.16)			0.87	(0.76,1.01)			1.10	(0.92,1.32)
South Atlantic			1.11	(0.96,1.27)			1.11	(0.95,1.30)			0.94	(0.81,1.09)			1.17	(0.98,1.41)
East South Central			1.19	(1.03,1.39)			1.21	(1.04,1.41)			0.98	(0.83,1.15)			1.43	(1.18,1.73)
West South Central			1.04	(0.90,1.21)			1.02	(0.88,1.19)			0.94	(0.81,1.09)			1.29	(1.06,1.56)
Mountain			1.22	(1.05,1.43)			1.11	(0.96,1.29)			1.14	(0.97,1.35)			1.44	(1.20,1.74)
Pacific			0.97	(0.84,1.11)			0.89	(0.76,1.03)			0.85	(0.73,0.99)			1.04	(0.88,1.24)
Individual Characteristics																
Mother’s Age (years)			1.01	(1.01,1.02)			1.01	(1.01,1.02)			1.01	(1.01,1.02)			1.01	(1.01,1.02)
Mother’s Race (ref: non-Hispanic white)																
non-Hispanic black			2.16	(2.07,2.25)			2.11	(2.02,2.20)			2.15	(2.06,2.24)			1.97	(1.88,2.06)
American Indian			1.16	(0.99,1.35)			1.00	(0.85,1.18)			1.30	(1.11,1.52)			1.20	(1.02,1.42)
Asian or Pacific Islander			1.13	(1.03,1.23)			1.03	(0.94,1.12)			1.15	(1.05,1.26)			1.14	(1.04,1.25)
Hispanic			1.08	(1.03,1.14)			0.99	(0.94,1.04)			1.10	(1.04,1.16)			1.10	(1.04,1.16)
Education (ref: less than HS)																
High School			0.98	(0.94,1.02)			0.94	(0.90,0.98)			0.92	(0.88,0.96)			0.95	(0.91,0.99)
Post-Secondary			0.76	(0.72,0.79)			0.73	(0.69,0.76)			0.72	(0.69,0.76)			0.74	(0.70,0.77)
Marital Status (ref: coupled)																
Single			1.26	(1.21,1.31)			1.23	(1.18,1.28)			1.24	(1.19,1.29)			1.25	(1.20,1.30)
Nativity (ref: born outside USA)																
US born			1.17	(1.12,1.23)			1.19	(1.13,1.25)			1.27	(1.21,1.33)			1.23	(1.17,1.30)
Birth Order (ref: first)																
Second			0.87	(0.83,0.91)			0.89	(0.85,0.92)			0.88	(0.85,0.92)			0.90	(0.86,0.94)
Third			0.88	(0.84,0.93)			0.94	(0.89,0.98)			0.89	(0.84,0.93)			0.91	(0.86,0.96)
Fourth or more			1.13	(1.08,1.18)			1.17	(1.12,1.22)			1.11	(1.06,1.16)			1.14	(1.08,1.19)

When adjusting for state-level and individual level covariates, income inequality was not significantly associated with an increased odds of infant (Table 3) or neonatal mortality (Table 4). However, the change in income inequality was significantly associated with an increased odds for both infant mortality (Table 3) and neonatal mortality (Table 4). For example, in 2007, for every increase in standard deviation of Gini coefficient from 1990 to 2007, was associated with a significant increase in odds for infant mortality (OR = 1.03, 95% CI = 1.00, 1.07) and neonatal mortality (OR = 1.05, 95% CI = 1.01, 1.09). Similar findings were observed in 2008, 2009, and 2010. Overall, results indicate that infants and neonates born in the US states that experienced a greater increase in income inequality since 1990 were more likely to die than those that were born in states with a smaller increase in income inequality.

The association between growing income inequality and infant mortality and neonatal mortality was observed among infants born of non-Hispanic white mothers but not non-Hispanic black mothers. When we stratified by mother’s immigrant status, the relationship between the change in the Gini Z-score and odds for infant mortality or neonatal mortality became insignificant with the exception of the year 2008. For an increase in one standard deviation of Gini from 1990 to 2008, there was a significant increase in infant mortality (OR = 1.04, 95% CI = 1.00,1.09) and neonatal mortality (OR = 1.07,95% CI = 1.01, 1.14) among US born mothers. When analyses were conducted among foreign-born mothers, an increase in Gini coefficient from 1990 to 2008 was associated with a higher likelihood of infant mortality (OR = 1.12, 95% CI = 1.03,1.21) and neonatal mortality (OR = 1.14, 95% CI = 1.04,1.25).

## Discussion

Using data on all live births and infant deaths in the United States between 2007 and 2010, we did not find a significant relationship between state-level income inequality and infant and neonatal mortality. However, we observed that those infants born in states that experienced the greatest growth in income inequality between 1990 and their year of birth, were more likely to die before their first birthday or within the first 28 days of life. When stratified by race, findings were observed only among infants born to non-Hispanic white mothers, but not infants of non-Hispanic black mothers. These findings indicate that growing inequality over time might have a detrimental influence on the health of newborn infants, but do not provide an explanation for the observed disparities in infant mortality risk by maternal race and nativity. Our results are consistent with those from an ecological study that identified as time increased, the effect of income inequality had an increasingly positive association with infant mortality rates [31].

Our findings add support to existing evidence that growing inequality can be detrimental to population health. For example, a meta-analysis has indicated that income inequality is associated with excess mortality [7]. However, when we stratified the analyses by race, our findings were only observed among non-Hispanic white Americans and not among infants with non-Hispanic black mothers. This is contradiction with previous work that has indicated income inequality is associated with premature mortality among non-Hispanic black adults [28]. Therefore, income inequality does not explain the white-black racial disparity in infant mortality risk and decreasing income inequality might not decrease the racial disparity in infant mortality. Other contextual factors, such as interpersonal and structural racism might play an important role in explaining the racial disparity in infant mortality.

This study might also provide evidence of the negative effects of the recession during the early 1990’s and the Great Recession in the late 2000’s. Income inequality increased during these economic downturns. Government responses to recessions sometimes include cutting social goods, such as welfare and health care, which can have detrimental effects on health directly, e.g., decreased access to health care, and indirectly, through an erosion of social cohesion. In particular, states are forced to contract services after a recession impacts resources. Therefore, residing in areas that experience a great increase in income inequality could be have led to an increased risk for infant mortality.

This study’s results should be interpreted in light of several limitations. First, the observed relationship might be due to endogeneity. Although we looked at change in income inequality over time, and therefore temporality could be addressed, endogeneity still persists due to our inability to control for all potential confounders. In other words, there could be residual confounding since potential confounders, such as individual level household income and other socioeconomic conditions were not available for this investigation. Another limitation is that data collected for this investigation were not intended for research. The quality of the data may not be accurate and therefore, might lead to misclassification. Nonetheless, the validity of the mortality data is very high. Also, infants with missing data and those whose state at birth and residence differ were excluded from the analyses. Those excluded might not be random and therefore, a selection bias might have been introduced. However, only a small percentage, 1.1%, of infants were excluded. Also, we investigated the race/ethnicity of mothers, and so most certainly included mixed race/ethnicity infants. We wished to examine race as a socially constructed determinant of health, and in the US, the mother’s race appears to be a stronger predictor of infant health than that of the father [32]. But we caution that income inequality might have a differing effect on mortality risk among mixed race/ethnicity infants. Finally, income inequality within smaller areas that are more proximal to the individual area-units, such counties and neighborhoods, might have a more influential role on infant mortality risk. We sought to build on previous work exploring state-level variations in income inequality. Future investigations should elucidate the role of income inequality within smaller residential areas on infant mortality risk.

Proposed pathways underlying the association between income inequality and infant mortality include a potential contextual mechanism, whereby inequality erodes social cohesion and underinvestment in social goods and programs [5]. Examples include public health, healthcare, education, and social welfare [5]. The lack of social services and social programs, might lead to adverse maternal health outcomes resulting in an increased risk for infant mortality [5]. Also, low social cohesion has shown to be detrimental to health, in particular among women [33]. Low social cohesion has been found to be associated with an increased risk for adverse mental health outcomes such as depression and stress [34], which all can be harmful to the infant’s health.

Another proposed mechanism is that when income inequality increases, individuals tend to make stressful comparisons with those around us, known as the relative deprivation theory [35]. In other words, when the income gap between individuals who interact with each other increases, the more stress and frustration is produced. Previous researchers have observed significant associations between these stressful comparisons and stress-related health outcomes such as smoking, obesity, diabetes, and adverse mental health outcomes [36]. However, we were not able to test the relative deprivation theory since the data necessary to conduct such analyses were not available in this cohort study. Nonetheless, stress and frustration generated from comparisons might have a detrimental effect on the health and well-being of mothers and their infants [37].

## Conclusions

We observed increasing state-level income inequality is a significant risk factor for infant mortality. More specifically, infants living in states with growing inequality were more likely to experience neonatal and infant mortality. However, the mechanisms in which income inequality lead to increased risk for neonatal and infant mortality have not been completely understood. Now that this association was found, future studies should determine whether this relationship is not only causal, but should begin to identify the mechanisms that link social factors to health outcomes. By doing so, public health professionals and policy makers may better prepared to develop and implement policies and interventions that could mitigate the harmful effects of income inequality.

## Data Availability

The datasets used and/or analysed during the current study are available in Centers for Disease Control and Prevention, National Center for Health Statistics (https://www.cdc.gov/nchs/nvss/index.htm).

## Abbreviations

AOR: Adjusted Odds Ratio
LBID: Linked Birth and Infant Death Records
NCHS: National center for health statistics
OECD: Organization for Economic Co-Operation and Development
OR: Odds ratio
SD: Standard deviation
